# Self-efficacy for writing and written text quality of upper secondary students with and without reading difficulties

**DOI:** 10.3389/fpsyg.2023.1231817

**Published:** 2023-09-22

**Authors:** Pär Sehlström, Christian Waldmann, Maria Levlin

**Affiliations:** ^1^Department of Language Studies, Umeå University, Umeå, Sweden; ^2^Department of Swedish, Linnaeus University, Växjö, Sweden

**Keywords:** self-efficacy for writing, reading difficulties, written text quality, upper secondary school, argumentative writing, poor word recognition, poor reading comprehension, L1/L2

## Abstract

**Introduction:**

Self-efficacy for writing (SEW) and reading ability are some of several factors that may be related to the quality of written text that students produce. The aim of the current study was (1) to explore the variation in SEW and written text quality in L1-Swedish and L2-English among upper secondary students with different reading profiles in L1 (typical reading vs. reading difficulties) and with different study backgrounds (SB1year or SB2years = one or two years of studies of Swedish and English, respectively), and in the next step (2) to explore if individual variations in L1-reading and SEW may explain variation in written text quality.

**Methods:**

Participants were 100 upper secondary students (aged 17–18) with different reading profiles operationalized as typical reading and reading difficulties. Data consisted of screening for word recognition and reading comprehension, text quality results from argumentative L1- and L2-writing tasks, school information on study background in Swedish/English, and students’ responses from an online survey about SEW.

**Results:**

As to SEW results, an ANOVA revealed significant main effects for reading profile and study background in L1, but in L2 there was only a significant main effect for reading profile. Written text quality results indicated that there was a significant interaction effect between reading profile and study background in L1, indicating that the significant main effect for reading profile on written text quality was influenced by the group of students with reading difficulties and SB1year. There was a significant main effect for reading profile and study background on written text quality in L2. Students with reading difficulties and SB1year were the most vulnerable group, and they had the lowest scores in L1/L2 SEW and written text quality in L1 and L2. Multiple regression results indicated that word recognition and SEW contributed significantly to L1-text quality, and word recognition, reading comprehension, and SEW contributed significantly to L2-text quality. Thus, this study sheds light on the under-researched area of L1/L2 SEW and text quality of students with reading difficulties at the level of upper secondary school.

**Discussion:**

Pedagogical implications are discussed and highlight the need for writing instruction across subjects in upper secondary school and for extra writing support/scaffolding for students with reading difficulties and shorter study background in the language subjects L1 (Swedish) and L2 (English).

## Introduction

There is a growing need for students to write well in L1 and L2 for participatory, educational, and professional purposes. In upper secondary school, students need to manage advanced levels of writing to be able to reach educational goals. Two factors that relate and contribute to writing performance are the writer’s reading ability and self-efficacy for writing (SEW), which relates to their beliefs about their own capability to perform a writing task (Bruning et al., 2013; Shanahan, 2016; Graham, 2020). However, the reciprocal relationships between reading ability, SEW, and writing performance are complex, and research findings are somewhat unclear. SEW has been found to be a strong predictor of written text quality in several studies included in Camacho et al.’s (2021) systematic review, whereas others have observed no association between SEW and written text quality (De Smedt et al., 2018, 2023).

Students with learning difficulties, which often include aspects of reading difficulties, may have lower self-efficacy in several domains (including writing) than peers without such difficulties (Saracoglu et al., 1989; Hampton and Mason, 2003; Baird et al., 2009; Klassen, 2010; Ben-Naim et al., 2017). In turn, perceived self-efficacy may affect if these students see a task as a manageable challenge or an obstacle (Stagg et al., 2018; Zumbrunn et al., 2020; De Busk-Lane et al., 2023). Although some studies report that reading difficulties may affect students’ SEW (Klassen, 2002a) and that students with reading difficulties have lower SEW than typical achievers (e.g., Slemon and Shafrir, 1997), other studies have found no difference between the two groups (Graham et al., 1993). In contrast, some research has suggested overly optimistic beliefs among students with reading difficulties (e.g., see Klassen’s overviews, 2002a,b).

As regards reading ability and written text quality, reading and writing are closely and reciprocally connected (Graham, 2020), and reading is considered a key resource which supports the composition of written text (Hayes and Berninger, 2014; Connelly and Dockrell, 2016). Similarly, the shared knowledge theory (Shanahan, 2016) assumes that reading and writing draw on similar sources, and the two skills can be viewed as “two buildings built on a common foundation” (p. 195). In the same vein, past scholarship has indicated that students’ reading difficulties may affect their writing performance in the sense that their reading difficulties may spill over on and compromise their writing (Berninger et al., 2008; Torrance et al., 2016; Kim, 2020). Several studies have revealed that students with word recognition difficulties and students with reading comprehension difficulties may struggle with L1 and L2 writing (Cragg and Nation, 2006; Herbert et al., 2020; Kormos, 2020; Graham et al., 2021; Sehlström et al., 2022). Although writing research is a burgeoning field of study, the writing of students with reading difficulties is under-researched (Berninger et al., 2008; Wengelin et al., 2014). Thus, there is a need to address this research gap, and, for instance, Graham et al. (2018a) “encourage writing researchers to include measures of reading in their studies” (p. 654). Especially at the level of upper secondary school, research is scarce.

To sum up, students with reading difficulties have been shown to have lower self-efficacy in many domains, but little is known about the SEW of upper secondary students with reading difficulties. Reading difficulties are of interest in this context as they may affect both SEW and written text quality, and SEW may, in turn, impact written text quality. Research findings are, however, not conclusive, and very little is known about the relationship between reading difficulties, SEW, and written text quality at the level of upper secondary school. Given the strong interconnection between reading and writing (Shanahan, 2016; Graham et al., 2018b; Kim, 2020) and the challenges that students with reading difficulties may face when writing (Graham et al., 2021), it is of particular interest to examine these students’ SEW (Schunk, 2003). This information could then be utilized to inform instruction, and to facilitate students’ reflections on their own writing, which is conducive to writing performance.

In this exploratory study, we investigate SEW and written text quality in Swedish (L1) and English (L2) in two groups of Swedish upper secondary students: one with typical reading and one with reading difficulties. To cater for the effect of length of study time and course complexity, study background in language subjects is included as a variable. Furthermore, we investigate how word recognition, reading comprehension, and SEW relate to written text quality in L1 and L2.

## Theoretical and empirical background

### Self-efficacy for writing and reading difficulties

The agentic and motivational concept of self-efficacy has been used in many fields to refer to metacognitive appraisals, which are domain-specific, future-oriented, and malleable (Klassen, 2002b; Botting et al., 2016; Schöber et al., 2018). Bandura (1997) conceptualizes self-efficacy as a person’s beliefs about their capabilities to accomplish a task successfully. If an individual has a slightly higher self-efficacy than ability, they may approach a demanding task with the view that it is a challenge within reach of their ability, and they will consequently be motivated to invest more time and effort. However, if an individual’s self-efficacy is low, they may regard the same assignment as something unachievable, which may result in making less effort or even giving up (Bandura, 1997; Carroll et al., 2009). On the other hand, too high self-efficacy in relation to ability may lead to an overestimation of one’s capability and to a simplistic approach not acknowledging the complexity of a task, which, in turn, may render simplistic or lower results. According to Bruning and Kauffman (2016), a person’s self-efficacy is shaped, among other things, by their experiences of performing a task successfully (enactive experiences), and by learning from observing others perform the same task (vicarious experiences). Also, emotional states, such as feeling good or anxious, and others’ feedback, suggestions, and encouragement may influence levels of self-efficacy. Generally, the self-efficacy of young students with reading difficulties tends to be low in several domains (Saracoglu et al., 1989; Ingesson, 2007; Klassen and Lynch, 2007; Baird et al., 2009; Ben-Naim et al., 2017). Many students with reading difficulties find aspects of metacognition challenging and they may be unaware of the importance of reflecting on aspects of knowledge and their own learning process, which is a cornerstone in metacognition (Klassen, 2002a, 2008; Butler and Schnellert, 2015). In the domain of writing, self-efficacy refers to students’ metacognitive perspectives and self-perceptions of their own writing ability. Some scholarship has found that students with reading difficulties tend to have lower self-efficacy for writing (SEW) than their typically achieving peers (Slemon and Shafrir, 1997), whereas other studies have indicated no differences in SEW between the two groups (Graham et al., 1993). Findings have also suggested that students with learning difficulties, which often include reading difficulties, have overly optimistic beliefs about their writing (Klassen, 2002a, 2008). Klassen’s (2002a) systematic review of students with learning difficulties and their SEW found that “five of six studies showed these students to overestimate their writing capabilities” (p. 97). A majority of participants were either younger students or university students.

The author states that there are several factors that underpin this unrealistic optimism. Firstly, self-efficacy is “construed as a form of metacognition” (Klassen, 2002a, p. 98). Students with learning difficulties often have problems with metacognition and metacognitive aspects of learning, which may partly be related to task misunderstandings and poor self-evaluation. Secondly, it is believed that these students have a more simplistic view of the actual writing process, whereas students without such difficulties have a more mature understanding of writing processes and task difficulty (Graham et al., 1993). Furthermore, responding to SEW tasks can be a challenge for students with learning difficulties as they have to process the statements and evaluate their own writing capacity in little time (Klassen, 2002a). Deficient estimation of SEW may lead to inappropriate strategies, faulty task understanding, and difficulties with self-regulating, including monitoring progress. Moreover, the findings of De Smedt et al. (2023) revealed that students who viewed writing as something innate and fixed, tended to eschew from revealing possible difficulties in writing, which, in turn, may be detrimental to their SEW.

Further, little is known about L2 SEW of students with reading and writing difficulties. Kormos and Nijakowska (2017) state that on top of “native language processing problems, students with specific learning difficulties often experience additional difficulties in acquiring additional languages […] Self-efficacy beliefs can have a powerful effect on both teachers’ and students’ actions and thoughts” (p. 31). Likewise, Ruegg (2018) discovered that strong SEW increases the chances of successful language learning and that structured teacher feedback on students’ L2 (English) writing enhanced students’ L2 writing self-efficacy. Leaving the specific focus on reading difficulties and SEW, we now turn to the reciprocal relationship between SEW and written text quality from a general perspective.

### Self-efficacy for writing and written text quality

Being able to reflect on one’s writing – strengths, challenges, self-regulation – is conducive to writing performance (Pajares, 2003; Knospe, 2017; Camacho et al., 2021). Previous research has revealed that SEW plays an important role for writing performance and written text quality (Shell et al., 1989; Pajares, 2003, 2007; Pajares et al., 2007a,b; Graham et al., 2018a; Camacho et al., 2021). Increased SEW is related to positive writing outcomes (Pajares et al., 2000; Bruning et al., 2013).

Past scholarship on SEW has mostly been undertaken by means of experimental studies, for example, interventions with pre- and posttests, or by means of correlational studies (Bruning and Kauffman, 2016). In one of the pioneering empirical studies looking into the relationships between SEW and performance, McCarthy et al. (1985) found that university students’ SEW explained about 15% of the variance in their writing scores on expository tasks. The study focused on writing mechanics in terms of composing an essay with no major spelling mistakes or run-on sentences. Similarly, several other findings indicate that SEW predicts students’ writing performance, including across grades (Shell et al., 1989; Pajares and Johnson, 1994; Pajares, 2003, 2007; Pajares et al., 2007b). In the same vein, Graham et al. (2018a) found that writing attitudes and SEW accounted for unique variation in text quality among their middle school students. The authors summarized what is known about the topic stating that SEW predicts individuals’ writing performance when it comes to measures designed by researchers after controlling for other factors such as reading, motivational beliefs, gender, poverty, and language proficiency.

Regarding the educational levels that previous research has investigated, studies have mostly focused on younger students and university students. For instance, in Camacho et al.’s (2021) systematic review of published, peer-reviewed articles between 2000 and 2018 covering grades 1–12, only 7 out of 62 samples included lower secondary or upper secondary students. Among several factors, the authors focused on the relationship between SEW and writing performance. Findings indicated that most studies found positive associations between SEW and writing performance. The systematic review also focused on grade level differences, but results are inconsistent, with some findings suggesting a decline in SEW in adolescence, and other findings suggesting an increase in adolescents’ SEW.

The early models for assessing SEW were unidimensional with only one factor catering for SEW, but later, Pajares (2007) conceived a two-factor model, which included basic skills and complex composition skills. More recently more fine-grained models have been designed (e.g., Bruning et al., 2013; Ekholm et al., 2015; Zumbrunn et al., 2016).

The influential self-efficacy for writing scale model of Bruning et al. (2013) included three non-hierarchical factors: (1) ideation, (2) writing conventions, and (3) self-regulation, i.e., management, monitoring, and evaluation. Henceforth, Bruning and colleagues’ self-efficacy for writing scale is referred to as SEWS, whereas the construct of self-efficacy for writing is referred to as SEW. Employing SEWS, the authors’ findings indicated that the three components of SEW – ideation, writing conventions, and self-regulation – were positively associated with text quality. In the same vein, meta-reviews have suggested that the three factors affect and account for variability in text quality in both L1 writing (Graham et al., 2018a; Camacho et al., 2021) and L2 writing (Sun et al., 2021). Many studies have employed Bruning et al.’s (2013) SEWS model. For instance, Soylu et al. (2017) found associations between SEW conventions and text quality in the form of American state assessment persuasive writing scores in an untimed writing session over a 2-day period regarding their upper secondary school sample. Zumbrunn et al.’s (2020) findings were similar for their mixed groups of elementary and lower secondary students, but the authors used an adapted version of SEWS. Associations have also been observed between SEW self-regulation and writing scores among Portuguese lower secondary students (Limpo and Alves, 2017), and between SEW content and text quality among Belgian upper elementary students (De Smedt et al., 2016). However, the latter study found no such relation between the other two factors and text quality. Similarly, the scores of Belgian students attending the academic track of upper secondary school revealed that there were no significant relations between students’ SEW and their argumentative text quality (De Smedt et al., 2023). However, writing is a complex activity, and De Smedt et al. (2023) reason that, in addition to SEW, other factors, such as language, basic writing skills, writing strategies, writing instruction and socio-economic status, may also contribute to text quality. As stated earlier, the studies under this subheading did not focus specifically on students with reading difficulties.

Less is known with respect to SEW in relation to L2 writing in English as a foreign language, especially as regards older students (Siekmann et al., 2023). Previous research has observed positive correlations between university students’ SEW and their L2 writing performance, and that SEW impacted writing performance more in L2 than in L1 (Sun et al., 2021, 2022). In the same vein, SEW has been found to predict both accuracy and complexity in university students’ narrative essays in L2 (Zabihi, 2018). Yet, findings are not conclusive (Siekmann et al., 2023), and, in terms of upper secondary students, research on these aspects is particularly scarce.

Scholarship also draws attention to the reciprocal aspects of self-efficacy perceptions and text quality (e.g., Pajares, 2007; Camacho et al., 2021). Pajares et al.’s (2000) study indicated that text quality contributed significantly to SEW. Similarly, Raoofi et al.’s (2017) group of university students with high writing proficiency had significantly higher SEW than the group with low-achieving peers. Thus, on the one hand, if a writer perceives writing as challenging, it is likely that their SEW is lower. On the other hand, making progress with one’s writing is not only about making progress with one’s writing skills and competence, but it is also about writing confidence. In other words, when facing a writing assignment, the perception of reality and one’s ability to reflect on the task at hand can decide and enhance sustained achievement motivation (Bouffard-Bouchard et al., 1991). Consequently, aspects of writing skills as well as aspects of SEW go hand in hand and need to be taken into account in parallel when exploring the complex relation between SEW and writing performance. As there is a close relationship between reading and writing, reading difficulties and written text quality are expanded on below.

### Reading difficulties and written text quality

With respect to the reciprocal reading – writing relationships, the shared knowledge theory (Shanahan, 2016) assumes that reading and writing draw on similar knowledge. Kim (2020) expanded this theory and developed the interactive dynamic literacy model which investigates the relation between reading and writing in greater detail. At the most basic level, Kim’s model can be likened to an iceberg whose tip is writing (spelling/written text production) and reading (decoding/written text comprehension). Below the surface are shared underlying emergent literacy, language and cognitive skills which make lexical-level (spelling/decoding) and discourse-level literacy (written composition/reading comprehension) possible. To develop lexical-level literacy skills, it is of paramount importance to be able to establish correct phonological, orthographic, and morphological representations. In turn, these emergent literacy skills depend on the underlying components of phonological processing skills (Melby-Lervåg et al., 2012) and morphological awareness (Rastle, 2022). Developing discourse-level literacy skills, on the other hand, depends on underlying components such as higher-order cognition and regulation, including inference-making, monitoring, goal setting, self-assessment and self-reinforcement, as well as foundational language skills (vocabulary and grammar) and discourse-level oral language (connected language). To conclude, both lexical-level and discourse-level oral language with their underlying components are a prerequisite for successful reading and writing. However, if one or both break down, reading comprehension and written composition will be affected negatively. In other words, considering that, to a certain extent, it is the same underlying component skills that affect reading and writing, it is not unexpected that students with reading difficulties also struggle with writing.

Students with word recognition difficulties have been found to struggle with spelling and lexical-level processing (Sumner et al., 2014; Wengelin et al., 2014; Torrance et al., 2016). Spelling difficulties tax working memory, partly for lack of automation and partly for avoidance strategies involving altering sentences to eschew words that are difficult to spell. In turn, these avoidance strategies and lack of automation may result in fewer cognitive resources available for discourse-level processing, for example, planning, conceptual development, text organization, and lexical and grammatical complexity (Wengelin, 2007; Wengelin et al., 2014; Torrance et al., 2016; Hebert et al., 2018; Sumner and Connelly, 2020). However, the transparency of the orthography of a language moderates the effect of poor word recognition, and, for instance, in shallow orthographies, students learn to spell and decode earlier. In contrast, these spelling and decoding skills are learnt later in deep orthographies, e.g., English (Seymour et al., 2003), which then may impact “the development of higher level processes, such as meaning-making processes in reading and writing” (Wengelin and Arfé, 2017, p. 29). Moreover, word recognition difficulties can also make it difficult for students to read through the text-written-so-far and detect what needs to be revised, which may be detrimental to their text quality (cf. Hayes and Berninger, 2014).

Students with reading comprehension difficulties have challenges with various levels of language, for example, words, sentences, and discourse (connected language), which in turn may affect and compromise their writing performance. Researchers agree that poor reading comprehension may have a negative impact on written text quality (Herbert et al., 2020; Kim, 2020). More specifically, students with poor reading comprehension have difficulties primarily at discourse-level, in such areas as text organization, for example, coherence, cohesion, cohesive devices (Cox et al., 1990; Cragg and Nation, 2006; Carretti et al., 2013, 2016; Re and Carretti, 2016; Sehlström et al., 2022), and lexical and grammatical complexity and syntactic diversity (Carretti et al., 2013, 2016; Re and Carretti, 2016). Content and conceptual development may be affected too. As to spelling, this group’s performance has almost been on a par with control groups (Cragg and Nation, 2006; Re and Carretti, 2016), but research has also found opposite results (Sehlström et al., 2022).

Writing in L2 adds an even greater cognitive challenge than writing in L1 for many students with reading difficulties (Kormos, 2012; Sehlström et al., 2022). Students with word recognition difficulties struggle with spelling due to lower automation levels of lexical-level skills. Deep orthographies, such as English, may take an extra toll on struggling spellers. Thus, these aspects may result in greater attention to formal aspects at the lexical-level at the expense of discourse-level processing (e.g., organization). Herbert et al. (2020) used the simple view of reading to define L1 and L2 groups with typical reading, poor word recognition, and poor comprehension in grades 4–6. The reading assessment of the L2 group – whose L1 was Portuguese, Punjabi, Tamil, Urdu, Chinese, and Russian – was carried out in their L2 (English). The results revealed that poor spelling, weak coherence and cohesion, and less complex language constituted the L2 writing features of the students with poor word recognition in L2. Similar effects were found concerning the subgroup with poor reading comprehension in L2, for example, poor coherence and cohesion, and less complex language. With respect to students with reading comprehension difficulties, in a recent Swedish study (Sehlström et al., 2022), it was found that the written text quality scores in L2 (English) of Swedish upper secondary students with reading comprehension difficulties in L1 were significantly below those of their peers with typical reading development, and especially challenging areas were discourse-level aspects such as cohesion and language use. However, in contrast to previous studies, spelling was significantly lower compared to the spelling levels of peers with typical reading. On the whole, though, scholarship on the effect of poor word reading or poor reading comprehension on older students’ L2 writing is scant (Herbert et al., 2020; Kormos, 2020; Sehlström et al., 2022).

To conclude, given that SEW is related to writing performance and that students with reading difficulties often are struggling writers, the relationship between reading ability and SEW is a fruitful avenue of investigation as both competence and confidence play a role in writing performance, especially in upper secondary school when reading and writing demands are high.

### The current study

This study investigates the text quality in argumentative writing and SEW in L1 (Swedish) and L2 (English) of upper secondary students with and without reading difficulties in L1. A factor that needs to be taken into account when exploring writing performance, is that students in Swedish upper secondary schools may have varying study backgrounds in the language subjects Swedish and English – the primary school subjects for explicit teaching of reading and writing. In Sweden, after the nine-year compulsory school including nine and six years of studying Swedish and English respectively, most students go on to the non-compulsory three-year upper secondary school attending vocational or higher education preparatory programs. The number of years that students then study L1-Swedish and L2-English is partly determined by the study program they attend. Higher education preparatory programs include a minimum of two years of Swedish and English. Most vocational programs include one year of Swedish and English, although some vocational students may opt for a second year too. The courses Swedish 1 and English 5 are studied in year 1, whereas the courses Swedish 2 and English 6 are studied in the second year. In other words, students study Swedish and English either only during year 1 (most vocational programs) or during years 1 and 2 (some vocational programs and all higher education preparatory programs). Thus, second-year upper secondary students who are the focus sample of the current study may have different study backgrounds in Swedish and English. However, all students have at least studied Swedish and English for a year, and by doing so, they have all had practice in writing argumentative texts, which are in focus in year one in upper secondary school. To account for the possible impact of differences in course study time and course complexity, study background is included as a variable in the study.

Against the above backdrop, and, as reading is a major resource for writing (Hayes and Berninger, 2014; Shanahan, 2016; Kim, 2020), it is fruitful to explore the quality of texts written by students with reading difficulties. This is especially true if one considers that between 15 and 20% of the population may find it difficult to read and comprehend texts (International Dyslexia Association, 2020). Little is known about the text quality in argumentative L1 and L2 writing and SEW of upper secondary students with reading difficulties, in particular in relation to variations in study background in the language subjects in school. The aim of this study is (1) to explore the variation in written text quality and SEW in L1-Swedish and L2-English among upper secondary students with different reading profiles in L1 (typical reading vs. reading difficulties) and with different study backgrounds in language subjects, and in the next step (2) to explore if individual variations in L1-reading and SEW may explain variation in written text quality. The research questions read:

1. What are the effects of reading profile and study background in language subjects on written text quality and self-efficacy for writing in L1 and L2?
2. To what extent can word recognition, reading comprehension, and self-efficacy for writing explain variation in written text quality in L1 and L2?

## Methods

### Participants

Participants were recruited from an upper secondary school located in a rural area in Sweden. One hundred and fifty-nine students (aged 17–18) constituted the total sample, of whom 100 students had a complete dataset regarding this study’s questions, reading, SEW, and text quality. The participants (*n* = 100) had Swedish as their first language. Fifty students were girls, and 50 students were boys. According to official statistics, the municipality’s unemployment rate is similar to that of the nation, and the annual median income is slightly below the national level, whereas the rate of citizens with a degree from post-upper-secondary education is more than 10% below that of the nation (Ekonomifakta.se, 2021).

Students were screened for word recognition (Olofsson, 1998) and reading comprehension (Järpsten and Taube, 2018). Means and SDs from the norm-referenced manuals have been used when calculating z-scores. Based on the screening outcome, students were divided into two reading profiles – students with typical reading (TR, word recognition and reading comprehension: *z* ≥ −0.59) and students with reading difficulties (RD, word recognition and/or reading comprehension: *z* ≤ −0.6). After attrition, there were 67 participants in the TR group (girls: 36, boys: 31) and 33 participants in the RD group (girls: 14, boys: 19). Forty-eight students attended higher education preparatory programs (TR = 37; RD = 11) and 52 students were in vocational programs (TR = 32; RD = 20). Based on the time participants had studied Swedish and English in upper secondary school, they were divided into two study background levels. Study background 1 year (SB1year) involves studies of Swedish and English in year one only, and study background 2 years (SB2years) indicates studies of Swedish and English during years 1 and 2. In Table 1, there is an overview of the participants in each reader subgroup, study background level and their reading scores in year 2.

**Table 1:** Means, standard deviations, and two-way between-groups analyses of variance exploring the effect of reading profile and study background for reading measures in Swedish (L1) year 2.

	Typical reading Mean (SD)		Reading difficulties Mean (SD)		F (η_p_^2^)		
	SB1year	SB2years	SB1year	SB2years	Reading profile	Study background	Interaction
	*n* = 10	*n* = 57	*n* = 11	*n* = 22			
Word recognition (z)	0.10 (0.45)	0.33 (0.62)	−1.20 (0.87)	−0.85 (0.56)	61.41*** (0.390)	3.34 (0.034)	0.15 (0.002)
Reading comprehension (z)	0.42 (0.57)	0.59 (0.56)	−0.18 (0.84)	0.04 (0.95)	10.74** (0.102)	1.24 (0.013)	0.89 (0.000)

**Significant effect at the *p* < 0.01 level. *** Significant effect at the *p* < 0.001 level. SB1year, study background 1 year, Swedish and English in year 1 only; SB2years, study background 2 years, Swedish and English in years 1 and 2.

Table 1 also presents a two-way between-groups analysis of variance to explore the impact of reading profile and study background on word recognition and reading comprehension in year 2. Since Levene’s test of equality of error variance indicated that the variance of the dependent variables was not equally distributed (word recognition, *p* = 0.018; reading comprehension, *p* = 0.043), a more stringent significance level (*p* < 0.01) was set. As expected, there was a significant main effect for reading profile on word recognition [*F* (3, 96) = 61.41, *p* < 0.001] and reading comprehension [*F* (3, 95) = 10.74, *p* = 0.001]. However, there were no significant main effects for study background on word recognition [*F* (3, 96) = 3.34, *p* < 0.07] or reading comprehension [*F* (3, 95) = 1.24, *p* = 0.27] with only small effect sizes. The interaction effect between reading profile and study background was neither significant for word recognition (*p* = 0.69) nor for reading comprehension (*p* = 0.89).

### Measures/materials

#### Word recognition in L1

##### Phonological decoding

Participants read triplets of pseudo-words silently and were then asked to mark the pseudo-word that sounded like a real word (Olofsson, 1998). The total number of correctly marked homophones within the time limit (2 min) was the total score.

##### Orthographic recognition

Participants read pairs of words silently (Olofsson, 1998). Each pair had one word that was spelled correctly, whereas the other one was a pseudo-homophone of the target word. The total number of correctly marked words within the time limit (2 min) made up the total score.

A composite measure of phonological decoding and orthographic recognition was used in this study with the internal validity 0.79 (Cronbach’s alpha).

#### Reading comprehension in L1

Participants were asked to read silently three factual texts (Järpsten and Taube, 2018). After each text, there was a multiple-choice task that tapped different literal aspects of the text as well as inferential content. Students had thirty-five minutes to complete the task. The total score was the sum of correct answers (maximum 21 points).

#### Written text measures in L1 and L2

The conceptual and structural design of the writing assignments was inspired by the Swedish and English language national writing assessment tests, which are set as timed tasks. The national writing assessment tests follow the form of summative writing assignments and are performed individually without collaboration or support/aid. Students wrote one argumentative text in L1-Swedish and L2-English, respectively, on two occasions. Students were instructed to take a stand on a suggestion from the principal at their school: School days should start at 10:00 am and end at 5:30 pm, and mobile phones should be banned during the whole school day.

Written text quality was examined using a slightly adapted version of Jacobs et al.’s (1981) analytic rating system covering seven commonly used categories in writing research: content, organization, cohesion, vocabulary, language use, spelling, and punctuation. The scale used in this study involved, as in the original version, four bands from very poor to excellent: 1 (*very poor*), 2 (*poor to fair*), 3 (*average to good*) and 4 (*very good to excellent*). To cater for a more fine-grained rating approach, half-marks were also awarded (1.5, 2.5, and 3.5). Detailed criteria were used to separate each band (see Supplementary Table 1 or Sehlström et al., 2022 for more information). In this study, a composite measure based on the outcome of the seven categories constituted text quality, and the scale’s internal consistency (Cronbach’s alpha) of the composite measure was very good for both the L1 (0.98) and L2 (0.97) texts. The texts were scored by two research assistants who were trained and blind to students’ reading profile and demographics. The interrater reliability was established through independent double-scoring of 20% of the texts. The intraclass correlation coefficients (ICC) were good for all seven aspects ranging from 0.76 to 0.92.

#### Self-efficacy for writing in L1 and L2

We measured participants’ SEW by using Bruning et al.’s (2013) well-established self-efficacy for writing scale (SEWS), which includes three SEW factors: ideation, writing conventions, and self-regulation. Prompts and the 16 SEW statements are listed in Supplementary Table 2. In line with much of past scholarship (Pajares et al., 2001; Bandura, 2006; Grenner et al., 2021; De Smedt et al., 2023), we employed a visual analog scale ranging from 0–100.

The original SEWS statements were translated into Swedish by the first author. To ensure the accuracy of the Swedish translation, the Swedish translation was translated back into English by a member of the research team (associate professor of English). Next, we piloted the Swedish version with eleven randomly chosen students and two teachers from a different upper secondary school in another municipality. The final version was adapted in accordance with pilot students’/teachers’ ideas and suggestions, which also included improvements of the layout. In the current study, SEWS had good internal consistency as the Cronbach’s alpha coefficient was 0.88 for SEWS in Swedish and 0.93 for SEWS in English. Students’ composite score of the three factors was used to indicate level of SEW.

### Procedure

The study was conducted in accordance with the Swedish Act relating to research involving humans (SFS 2003:460, 2003) and the ethics guidelines of the Swedish Research Council (Stafström, 2017). Prior to data collection, the school’s principals and teachers gave their oral consent and students gave their written consent to participate in the study. Word recognition and reading comprehension were assessed in groups of 30–50 students during three separate sessions by a member of the research team or by a teacher at the school in the spring semester of the second year of their three-year voluntary upper secondary school program. Tasks that measured word recognition and reading comprehension were administered and scored according to the standard procedures in the manuals (Olofsson, 1998; Järpsten and Taube, 2018). The writing assignments were carried out in the form of two separate impromptu writing sessions at the students’ school in groups of 30–50 students, also during their second year. Students wrote one of the argumentative assignments in Swedish and the other in English. The order of language and assignment was counterbalanced in a Latin square design. They had 45 min to write their texts using the Scriptlog keystroke-logging software (Frid et al., 2014) on their laptops. The process data have not been investigated in this study, but they have been examined in other studies. No spelling aids or dictionaries were allowed. Three weeks after their last writing session, students filled in the web survey about self-efficacy for L1- and L2-writing.

### Data analyses

A two-way between groups ANOVA was used to explore the effects of reading profile (typical reading vs. reading difficulties) and study background (1 year of Swedish/English vs. 2 years of Swedish/English) on written text quality and self-efficacy in L1 and L2 (RQ1). Analyses of skewness revealed values between −0.41 and  −0.96 for the dependent measures in the ANOVA. No extreme outliers were identified in the boxplots. Levene’s test for the dependent measures was non-significant, all *p*-values >0.29, indicating equal variance across groups for all the dependent measures. The significance value was set at *p* < 0.05 for all comparisons. Effect sizes for the ANOVA are reported as partial eta squared (η_p_^2^; small effect = 0.01, medium effect = 0.06 and large effect = 0.138, Cohen, 1988).

Multiple regression analysis was used to explore to what extent word recognition, reading comprehension and self-efficacy for writing can explain variation in written text quality in L1 and L2 (RQ2). No extreme outliers were identified in the boxplots among the dependent or independent variables. The significance value was set at *p* < 0.05.

## Results

### The effects of reading profile in L1 and study background on L1 text quality and SEW

The descriptive statistics for text quality and self-efficacy for writing in L1 and the results of the two-way between-groups ANOVA are presented in Table 2.

**Table 2:** Means, standard deviations, and two-way between-groups analyses of variance exploring the effect of reading profile and study background on written text quality and self-efficacy for writing in Swedish (L1).

	Typical reading Mean (SD)		Reading difficulties Mean (SD)		*F* (η_p_^2^)		
	SB1year	SB2years	SB1year	SB2years	Reading profile	Study background	Interaction
	*n* = 10	*n* = 56	*n* = 11	*n* = 22			
Text quality	2.12 (0.68)	2.58 (0.54)	1.28 (0.52)	2.54 (0.45)	10.79** (0.102)	40.16*** (0.297)	8.52** (0.082)
Writing self-efficacy	61.36 (14.29)	65.63 (21.62)	35.76 (24.38)	60.84 (26.14)	7.22** (0.070)	6.75* (0.066)	3.39 (0.034)

*Significant effect at the *p* < 0.05 level. **Significant effect at the *p* < 0.01 level. ***Significant effect at the *p* < 0.001 level. SB1year, study background 1 year, Swedish and English in year 1 only; SB2years, study background 2 years, Swedish and English in years 1 and 2.

**Figure 1:**
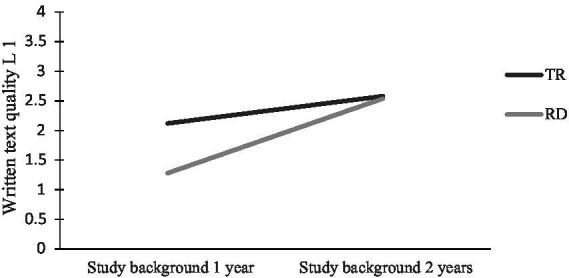
Written text quality in L1 for students with different reading profiles and study backgrounds in language subjects.

Generally, students with SB1year scored lower in text quality than peers with SB2years, and SB1year-students with RD received the lowest text quality scores of all groups. There was a significant interaction effect (*p* = 0.004) between reading profile and study background in Swedish, indicating that the main effects for reading profile [*F* (1, 95) = 10.79, *p* = 0.001] and study background [*F* (1, 95) = 40.16, *p* < 0.001] were influenced by the group of students with reading difficulties and study background 1 (see Figure 1).

**Figure 2:**
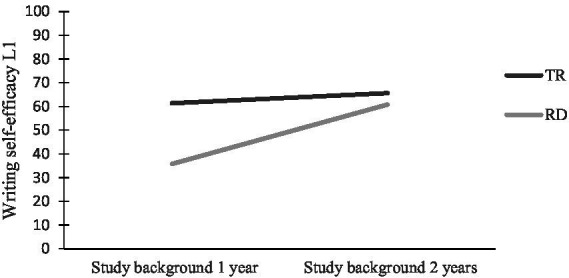
Self-efficacy for writing in L1 for students with different reading profiles and study backgrounds in language subjects.

As to SEW, the general pattern is that students’ level of reading skills plays a role for their SEW, as does study background; SB1year-students with RD had the lowest score of all groups. More specifically, there were no significant interaction effects (*p* = 0.07) between reading profile and study background in relation to writing self-efficacy. There was a statistically significant main effect for reading profile [*F* (1, 96) = 7.22, *p* = 0.008] and for study background [*F* (1, 96) = 6.75, *p* = 0.011] with medium effect sizes (partial eta squared: reading profile = 0.070; study background = 0.066) (see Figure 2).

### The effects of reading profile in L1 and study background on L2 text quality and SEW

Table 3 shows the descriptive statistics for text quality and self-efficacy for writing in L2 and the results of the two-way between-groups ANOVA.

**Table 3:** Means, standard deviations, and two-way between-groups analyses of variance exploring the effect of reading profile and study background on written text quality and self-efficacy for writing in English (L2).

	Typical reading Mean (SD)		Reading difficulties Mean (SD)		F (η_p_^2^)		
	SB1year	SB2years	SB1year	SB2years	Reading profile	Study background	Interaction
	*n* = 10	*n* = 54	*n* = 11	*n* = 21			
Text quality	2.22 (0.54)	2.85 (0.58)	1.58 (0.74)	2.33 (0.68)	13.77*** (0.130)	19.40*** (0.174)	0.13 (0.001)
Writing self-efficacy	59.11 (16.72)	60.05 (24.70)	33.83 (24.32)	50.86 (29.46)	7.38** (0.071)	2.01 (0.021)	1.61 (0.016)

**Significant effect at the *p* < 0.01 level. ***Significant effect at the *p* < 0.001 level. SB1year = study background 1 year, Swedish and English in year 1 only; SB2years = study background 2 years, Swedish and English in years 1 and 2.

**Figure 3:**
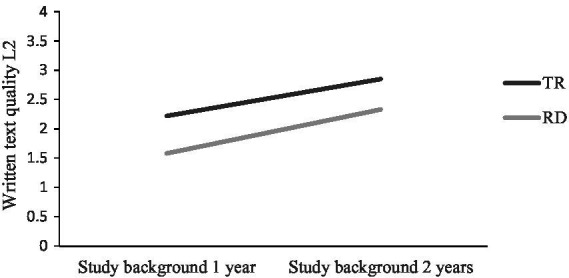
Written text quality in L2 for students with different reading profiles and study backgrounds in language subjects.

A general observation is that both groups with SB2years performed better than their peers with SB1year when it comes to L2-text quality. The lowest text quality scores of all groups were observed in the SB1year-group with RD. There were no statistically significant interaction effects between reading profile and study backgrounds for text quality (*p* = 0.717) and writing self-efficacy (*p* = 0.208). There was a statistically significant main effect for reading profile [*F* (1, 92) = 13.77, *p* < 0.001] and study background [*F* (1, 92) = 19.40, *p* < 0.001] with large effect sizes (partial eta squared: reading profile = 0.13; study background = 0.17). A visualization of L2-text quality scores can be seen in Figure 3.

**Figure 4:**
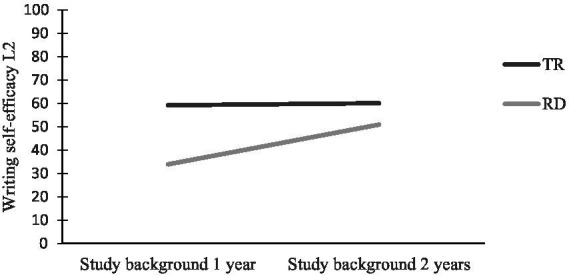
Self-efficacy for writing in L2 for students with different reading profiles and study backgrounds in language subjects.

For writing self-efficacy, the global picture indicates the highest scores among both groups with typical reading regardless of study background, followed by the two SB1year-groups. As can be seen in Figure 4, writing self-efficacy was lowest for the SB1-group with RD. There was a significant main effect for reading profile [*F* (1, 96) = 7.38, *p* = 0.008], with medium effect size (partial eta squared = 0.07), but there was no main effect for study background [*F* (1, 96) = 2.01, *p* = 0.160].

### Relations between reading skills, SEW, and text quality in L1 and L2

Table 4 shows to what degree variation in text quality in L1 and L2 can be explained by word recognition, reading comprehension, and SEW. The total variance in text quality in L1-writing explained by the model was 25%, *F* (3, 94) = 11.81, *p* < 0.001. The individual predictors were examined further and indicated that word recognition (*p* = 0.010) and writing self-efficacy (*p* = 0.002) were the only significant predictors of text quality in argumentative L1-writing.

**Table 4:** Regressions predicting written text quality in Swedish (L1) and English (L2).

	Beta	*t*	*P*	*F*	df	*p*	adj.R^2^
Written text quality in L1							
Overall model				11.81	3,94	<0.001	0.25
Word recognition in L1	0.25	2.67	0.010				
Reading comprehension in L1	0.15	1.58	0.117				
Writing self-efficacy in L1	0.30	3.13	0.002				
Written text quality in L2							
Overall model				29.10	3,91	<0.001	0.47
Word recognition in L1	0.32	3.92	<0.001				
Reading comprehension in L1	0.35	4.32	<0.001				
Writing self-efficacy in L2	0.28	3.50	<0.001				

The total variance in text quality in L2-writing explained by the model was 47%, *F* (3, 91) = 29.10, *p* < 0.001. The individual predictors were examined further and indicated that word recognition, reading comprehension, and writing self-efficacy all contributed significantly (*p* < 0.001) to the quality in argumentative L2-writing.

## Discussion

In the current study, research question 1 focused on exploring the effect of reading profile (reading difficulties, RD vs. typical reading, TR) and study background (SB1year vs. SB2years) in Swedish (L1) and English (L2) on the outcome variables written text quality and self-efficacy for writing (SEW) in Swedish and English. Research question 2 focused on testing if a model that included word recognition (L1), reading comprehension (L1), and SEW (L1/L2) as predictor variables, reached significance in explaining the variance in written text quality in argumentative tasks in Swedish and English.

### Reading profile and study background effects on written text quality in L1 and L2

In regard to L1 and L2 written text quality, the general picture is that all four subgroups produced texts within the lower bands (very poor or poor to fair) regardless of reading profile and study background. Students with typical reading and two years of study background in Swedish and English in upper secondary school (SB2years) performed best among the subgroups. However, there were also individual variations within the groups, and although the group means were relatively low, there were individuals performing well. These findings indicate that argumentative writing was challenging for most students in the study and findings are in line with previous research (Ferretti and Lewis, 2013; Traga Philippakos and MacArthur, 2020). In the same vein, educational statistics (National Center for Education Statistics, 2012) have revealed low scores for the majority of upper secondary students’ argumentative texts in American large-scale assessments. Landrieu et al. (2022) conclude that the “argumentative writing proficiency of students appears to be highly substandard” (p. 2). However, the global picture apart, there are interesting group differences that are worth scrutinizing.

Zooming in on L1 text quality, the group with reading difficulties and SB2years performed on a par with the group with typical reading and the same study background. This suggests that reading difficulties may not imply extra challenges in writing for those students who opt for a second year of studying Swedish and English in upper secondary school. In contrast, students with RD and SB1year in Swedish and English had significantly lower text quality compared with peers with TR and the same study background. Thus, in the group with SB1year, reading difficulties seem to tax text quality severely (cf. Figure 1 where interaction effects were observed). It is difficult to pinpoint what this difference between SB1year and SB2years depends on in relation to the students with reading difficulties. The results are probably due to a combination of reading difficulties and study program effects. Past research (Westman, 2009; Sturm, 2016) and statistics of national assessments in writing (SNAE, 2017a,b) indicate that students attending vocational programs find writing challenging, and so do students with reading difficulties (Graham et al., 2021). For instance, significant differences have been found in text quality between groups with reading difficulties and controls (Cragg and Nation, 2006; Carretti et al., 2013; Torrance et al., 2016). These studies have indicated that students with reading difficulties often struggle with coherence, cohesion, content, and mechanics, apart from writing texts of lower linguistic complexity when it comes to grammar and vocabulary (Wengelin, 2007; Carretti et al., 2013; Wengelin et al., 2014; Torrance et al., 2016; Sumner and Connelly, 2020). Previous studies have been carried out in elementary school (Carretti et al., 2013), lower secondary or upper secondary school (Wengelin et al., 2014; Torrance et al., 2016), or at university level/adults (Wengelin, 2007; Sumner and Connelly, 2020). Our study adds to the extant literature by showing that there may be an interaction between reading difficulties and study background at the level of upper secondary school. This interaction may then have the concomitant effect of reading difficulties making writing more challenging for students attending a vocational program with fewer courses in language subjects.

Regarding L2-English written text quality, there was a main effect for reading profile as well as for study background with no significant interaction effects. In other words, reading difficulties imply greater difficulties in writing a good text compared with peers with typical reading. Moreover, students with SB2years wrote better texts compared with students with SB1year (see also Figure 3). These findings are consistent with previous research, which has revealed that writing in L2 is challenging for students with reading difficulties identified in L1 or L2 (Herbert et al., 2020; Levlin et al., 2022; Sehlström et al., 2022). The study design of the earlier-mentioned studies varied in several aspects regarding age group, genre, and if reading difficulties were identified in L1 or L2. For example, Herbert et al. (2020) focused on students identified with reading difficulties in their L2 and students in grades 4–6, while Levlin et al. (2022) focused on students with reading difficulties in L1 and long-term effects on reading and writing in L2. In Levlin et al. (2022), students identified with reading difficulties in L1 in early elementary school performed low scores in the L2-writing part of the national assessment test in grade 9. In another study by Sehlström et al. (2022), it was found that upper secondary students with reading comprehension difficulties in L1 scored significantly below peers with TR in such categories as cohesion, language use, and spelling when writing in L2. The current study adds to previous studies by confirming that L2 writing continues to be challenging for students with reading difficulties in upper secondary school, and this is also the case for students attending higher education preparatory programs with more courses in language subjects.

There may be several reasons for students with reading difficulties in L1 having challenges with L2-writing. First, writing in an L2 adds an extra cognitive strain, by putting a greater load on working memory (Kormos, 2012), and this will probably take a heavy toll on students with reading difficulties since limited capacity in working memory is quite common (see overview in Cain (2022)). Second, many of these students have linguistic difficulties in L1 related to vocabulary, grammar, and discourse (connected language) (see overview in Cain (2022)), which in turn may impact translation of ideas into language in both L1 and L2 writing (Sehlström et al., 2022). Writing in L2 implies that the translation process may lead to having to translate from L1 into L2 too. It will then vary between individuals how challenging the translation process will be as it may depend on their linguistic experience in L2 (Lindgren et al., 2008). Third, spelling is another challenge for many students with reading difficulties. English is a very opaque orthography – more so than Swedish. This fact may accentuate the secondary effects of spelling difficulties making students focus on lexical-level processing and missing out on global aspects such as discourse-level processing (e.g., coherence). In the current study, the students with reading difficulties had to a greater extent poor word recognition rather than poor reading comprehension at a group level. Thus, it can be deduced that aspects of the lexical-level skills may have been particularly challenging for many of the participants.

There was also a main effect for study background in relation to text quality in L1 and L2. Students with SB1year performed below students with SB2years, regardless of reading profile (see Figures 1, 3). One factor could be differences in time on task between the two study backgrounds, as more time on task is generally conducive to written text quality (Wengelin and Arfé, 2017). Students with SB1year had not studied Swedish and English during their second year when they wrote their essays – in contrast to the students with SB2years. All students with SB1year attended vocational programs. National statistics evidence that many upper secondary students struggle with L1 and L2 writing and especially so students in vocational programs. In the 2017 national assessment tests in Swedish and English, 16% of students failed to meet the knowledge requirements for writing in Swedish, and the corresponding figure for English was 7% (SNAE, 2017a,b). Failure rate specifically for vocational programs was 28% for writing in Swedish and 15% for writing in English. Thus, it is no surprise that students with SB1year had the lowest writing performance. Consequently, this group is the most vulnerable when it comes to L1 and L2 writing.

Another explanatory factor could be that self-selection and tactical choices are at work here (Edvardsson and Bruce, 2023). For instance, some students with reading difficulties may have avoided programs that include Swedish and English in year 2, whereas others may have opted for Swedish and English years 1 and 2 despite their reading difficulties due to better coping strategies. There may be many factors which affect the choice of study program, and it is difficult to express any certainty about the different factors that may have influenced students’ choices in the current study. Students with reading difficulties and SB2years had basically similar reading levels as their RD-peers with SB1year. However, we do know that, for instance, SES-factors and parents’ educational background may influence individuals’ study choices (Korat and Schiff, 2005; Watson et al., 2016; Gil et al., 2019). These aspects were not possible to explore on an individual level in this study as no such data were available.

### Reading profile and study background effects on self-efficacy for writing in L1 and L2

Since self-efficacy for writing (SEW) has been found to be related to writing performance (Pajares, 2003; Bruning et al., 2013; Graham et al., 2018b) and there is scarce knowledge about how reading difficulties may relate to SEW, our study also explored the effect of reading ability on students’ SEW. The results revealed that reading profile in L1 had a significant main effect on SEW in both languages. Furthermore, study background had a significant main effect on SEW in L1 but not in L2. In other words, how long students had studied Swedish and English played a role for their SEW in L1 but not for their SEW in L2 in the current study.

Past research has indicated that adult university students with reading difficulties have lower self-efficacy than peers without such difficulties (Slemon and Shafrir, 1997; Stagg et al., 2018). The current study confirms the same SEW-patterns among upper secondary students with reading difficulties. Low SEW-scores in both L1 and L2 of our upper secondary students with reading difficulties, can also be seen in light of older students developing and deepening their skills in understanding and analyzing the complexity of tasks and skills, as opposed to younger students’ generally strong self-efficacy with little differentiation between tasks (Klassen, 2002a; Pajares, 2007; Muenks et al., 2018; Grenner, 2021). Furthermore, the students with reading difficulties in L1 were challenged in their writing in both L1 and L2, and that may be reflected in a decreased SEW in L1 and L2. Some previous studies have found that students with learning difficulties overestimate their SEW (Graham and Harris, 1989a,b; Klassen, 2002a,b, 2008). However, the current study does not confirm that pattern. The differences in outcome may be due to a focus on younger students in previous studies (Graham and Harris, 1989a,b; Klassen, 2002a,b, 2008).

Our investigation also sheds light on the effect of study background in language subjects on SEW. The SB1year-group with reading difficulties had the lowest scores on SEW in L1 (approaching significant interaction effects, see Figure 2) and in L2. This outcome suggests that these students have particularly low confidence in performing writing tasks. This group’s low SEW is in line with their very low text quality results. Thus, reading difficulties in combination with little time on task and writing instruction are an unfortunate combination in terms of SEW and written text quality.

One explanation for the study background effects in L1 could be self-selection related to other factors than reading and writing performance. We did not find that the students with reading difficulties and SB1year had greater reading difficulties than their peers in the SB2years group (see Table 1). We cannot be certain about cause and effect as SEW and writing performance work reciprocally (Pajares et al., 2000; Camacho et al., 2021). For instance, we do not know whether SB1year-students with reading difficulties in the first place chose their programs and subjects because of low SEW or because of low reading and writing performance. The relatively higher L1SEW estimation of the SB-2 year group with reading difficulties may be related to students’ time on task. It is reasonable to believe that their literacy studies (Swedish and English) in the second year have enhanced both their writing and metacognitive skills. The result could also be related to higher SEW from the beginning, before applying to upper secondary school. To conclude, our findings suggest that reading difficulties in combination with attending an upper secondary school program with little focus on language subjects are related to lower L1 SEW. With respect to L2, reading profile in L1 was related to SEW in L2. Once again, however, the group with the combination of study background one and reading difficulties was the most vulnerable group in terms of having the lowest confidence in writing in L2 (see Figure 4).

### Factors explaining the variance in written text quality in L1 and L2

As to L1 written text quality, a regression model including word recognition, reading comprehension, and SEW explained in total 25% of the variation in text quality, with SEW and word recognition contributing significantly. This is in line with several of previous studies revealing an association between SEW and written text quality (De Smedt et al., 2016; Limpo and Alves, 2017; Soylu et al., 2017; Zumbrunn et al., 2020), albeit findings not being conclusive (De Smedt et al., 2018, 2023). Our results are also congruous with past scholarship which has suggested associations between lower-level transcription skills and written text quality (Graham and Santangelo, 2014; Limpo et al., 2017). It is well known that lexical-level skills may influence overall text quality (Berninger et al., 2002; Dockrell, 2009; Limpo and Alves, 2013; Sumner et al., 2014; Hebert et al., 2018; Kim, 2020) in elementary grades when spelling and word recognition are not yet automatized. The current study suggests that lexical-level skills (word reading) relate to general written text quality also at the level of upper secondary school even in a semi-transparent orthography as Swedish. This is probably due to the complex interaction between word recognition and spelling, and word recognition influencing overall text quality through spelling. As Kim (2020) describes in the interactive dynamic literacy model there is a strong association between reading and spelling on the lexical-level. For instance, underlying phonological processing skills are at work when reading and writing. Reading involves decoding words’ phonological identity from written words, while writing involves encoding phonological information into written words. In addition, levels of word recognition proficiency may also have secondary effects on written text quality, by influencing the process of reviewing and revising the text-written-so-far (cf. Hayes and Berninger, 2014).

Contrary to the little research that exists (Cragg and Nation, 2006; Carretti et al., 2013), we observed little association between reading comprehension and written text quality in L1. This was not an expected outcome, since writing an argumentative task demands quite advanced vocabulary, grammar and discourse-level processing, as does reading comprehension (Kim, 2020). There may be several reasons for reading comprehension not contributing to text quality in the current study. It could be that the argumentative task did not require our students to engage in reading complex source materials, which might have added an extra cognitive load taxing reading comprehension. It could also be that the tasks focused on fairly everyday matters of a less complex nature demanding fewer aspects for ideational development, which, in turn, may have led to students not having to manage and use complex concepts and grammar.

With respect to L2-English written text quality, a regression model including word recognition (L1), reading comprehension (L1) and SEW (L2) explained in total 47% of the variation in text quality. Word recognition, reading comprehension and SEW contributed significantly to written text quality. Thus, reading comprehension in L1 proved to be significant for the variation in text quality in English, but not in Swedish. One likely explanation for this difference is the combination of the extra cognitive load that L2-writing involves and the cognitively taxing argumentative genre, which puts high demands on rhetorics when it comes to text organization and linguistic complexity, particularly so in a foreign language. These two aspects may explain why the comprehension component is important in this context. More specifically, although one would expect these aspects to impact Swedish (L1) text quality too, it is an even greater challenge to tackle these aspects successfully in L2 as students also have to translate their ideas into linguistic content in L2 (Lindgren et al., 2008). Thus, one can compare with Kim’s (2020) model explained earlier and the interaction between reading and writing at the discourse-level.

To conclude, the findings of the current study indicate there is an association between reading in L1 and L1 and L2 text quality, which in turn lends support to and corroborates the shared knowledge theory (Shanahan, 2016) and Kim’s (2020) interactive dynamic literacy model mentioned earlier. Both these theoretical frameworks assume that reading and writing share the same underlying linguistic proficiency. Phonological processing skills affect both word reading and spelling on a lexical level, and oral language and higher order cognitive skills affect reading and writing on a discourse level. Further, universal text attributes such as knowledge about characteristics of text, genre and rhetorics, may affect both reading and writing on discourse-level. Especially in upper secondary school, there are greater demands on language and higher order skills which may put constraints on writing performance on discourse-level. At this advanced level, students’ writing is more concerned with the more complex knowledge-transforming instead of the more basic knowledge-telling (Bereiter and Scardamalia, 1987).

When it comes to SEW, the current results are consistent with past studies, which have indicated a relationship between SEW and written text quality (Pajares et al., 2007b; Bruning et al., 2013; Zumbrunn et al., 2020). In the same vein, Villalón et al. (2015) state that SEW predicts high schoolers’ writing performance more consistently than other motivational factors (writing apprehension, perceived value of writing) or self-belief (academic self-concept). However, earlier studies have used a wide range of measures for high schoolers’ text quality (i.e., the outcome measure) in relation to SEW: (1) self-reported writing grades and statewide writing assessment scores (Bruning et al., 2013), (2) language arts teachers’ estimation (rating) of students writing competence (Pajares et al., 2007b), and (3) high schoolers’ ELA grades (Zumbrunn et al., 2020). The latter authors call for more studies that include scored samples of students’ writing performance. Our study addresses the research gap and reveals that SEW also contributes to L1 written argumentative text quality also if text quality is based on manually scored samples at the level of upper secondary school. In regard to SEW in L2, few studies have investigated the relation between SEW and writing performance in L2. The little research that exists has mostly focused on younger students or university students and has found significant correlations between L2 SEW and L2 writing performance (Sun et al., 2021, 2022). Our findings contribute to the field by revealing associations between SEW and text quality in L2 also at the level of upper secondary school.

### Limitations and future research

The interpretation of the results should be seen in light of a few limitations. First, our students wrote one argumentative task in Swedish (L1) and English (L2). Several tasks in the same genre in the same language would have allowed for greater generalizable claims (van Steendam et al., 2012). Thus, future research would benefit from heeding this advice, if possible. However, one has to bear in mind that writing several tasks may lead to fatigue for a special population that may find it burdensome to write. Thus, the risk for writing fatigue was the reason for our methodological decision to have students write one text in each language. Also, we did not want to intrude on students’ timetable too much. Second, we did not have any information on how much writing instruction students received and the instructional context, which is a factor that relates to performance. Consequently, future studies should include information on writing instruction too. Third, as students’ literacy contexts/habits influence their reading and writing, it would be fruitful to include contextual aspects such as students’ reading and writing habits in relation to students’ written text quality and SEW in future writing research. Fourth, in our study, students were not allowed to use any aids when writing their texts. Past research has shown that appropriate assistive technology may enhance the self-efficacy of students with reading and writing difficulties (Rousseau et al., 2017; Camacho et al., 2021). Thus, it would be of interest to investigate the effect of technological aids on upper secondary students’ SEW and text quality by using an intervention study design.

## Conclusion

A central finding is the especially weak text quality in L1 and L2 of students with reading difficulties and only one year of studies in upper secondary school in the language subjects Swedish and English. Reading plays a role for both L1 and L2 writing performance and covaries with study background. In other words, both reading ability and how long one had studied Swedish and English affected the outcome. Regarding L1-SEW, both reading profile and study background affected the outcome, while L2-SEW was affected only by reading profile. Word recognition was a significant predictor of L1 text quality, whereas both word recognition and reading comprehension were significant predictors of L2 text quality. SEW contributed significantly to written text quality in both L1 and L2. Thus, overall findings suggest lending support to the assumption that reading is a resource for writing (cf. Hayes and Berninger, 2014), and to the theoretical frameworks of the shared knowledge theory (Shanahan, 2016) and the interactive dynamic literacy model (Kim, 2020), which assume that reading and writing share the same underlying proficiency.

Our results highlight the need to give extra writing support/scaffolding in L1 and L2 to students with reading difficulties (especially with study background one year in language subjects in vocational programs). Considering the importance of writing for educational attainment at this level, the overall poor outcome of argumentative text quality regardless of reader subgroup underscores the need to give coherent form to writing instruction in all subjects across the curriculum in upper secondary school.

As it is of interest for teachers to understand students’ own views of their writing challenges for feedback and feedforward, students’ SEW reports can be used in writing instruction to improve the quality of such feedback/feedforward by teachers (or by peers). In other words, students’ own SEW statements can help students put their own thoughts about their writing into words, which can help teachers identify each individual’s perceived writing strengths and challenges (ideation, writing conventions, self-regulation etc.) and consequently give students appropriate and effective support/scaffolding. This approach may then also facilitate students’ reflections on their writing and meta-discussions about writing in the school context, as it may enhance/scaffold students’ own meta-language to talk about their writing.

## Data availability statement

The datasets presented in this article are not readily available because of ethical considerations. Requests to access the datasets should be directed to the corresponding author.

## Author contributions

All authors listed have made a substantial, direct, and intellectual contribution to the work and approved it for publication.

## Funding

This work was supported by the Swedish Research Council (grant number 2018-03729).

## Conflict of interest

The authors declare that the research was conducted in the absence of any commercial or financial relationships that could be construed as a potential conflict of interest.

## Publisher’s note

All claims expressed in this article are solely those of the authors and do not necessarily represent those of their affiliated organizations, or those of the publisher, the editors and the reviewers. Any product that may be evaluated in this article, or claim that may be made by its manufacturer, is not guaranteed or endorsed by the publisher.
