# Supplementary material for: Self-efficacy for writing and written text quality of upper secondary students with and without reading difficulties
Source: Front Psychol. 2023 Sep 22;14:1231817. doi: 10.3389/fpsyg.2023.1231817 (PMC10557487; doi:10.3389/fpsyg.2023.1231817)
Supplement: Supplementary file 1 [file Table_1.docx]

# Supplementary Table 1

*Criteria for Each Measure in the Adapted Version of Jacob et al.’s (1981) Analytic Scoring Scheme*

| Measure | Band | Criteria |
| --- | --- | --- |
| Content | 4 | Knowledgeable, substantive, thorough development of thesis, relevant to assigned topic. |
|  | 3 | Some knowledge of subject, limited development of thesis, mostly relevant to topic. |
|  | 2 | Limited knowledge of subject, inadequate development of topic |
|  | 1 | Does not show knowledge of subject, non-substantive. Or not enough to evaluate. |
| Organisation | 4 | Ideas clearly stated. Succinct, well-organised, logical sequencing of ideas. |
|  | 3 | Somewhat loosely organised, but main ideas stand out. Logical but occasionally incomplete sequencing of ideas. |
|  | 2 | Ideas confused or disconnected. A lack of logical sequencing of ideas. |
|  | 1 | No organisation. Or not enough to evaluate. |
| Cohesion | 4 | Very good use of cohesive devices with a wide range of cohesive devices. Arguments are linguistically connected in a smooth way. |
|  | 3 | A satisfactory use of cohesive devices, simple at times but no strain on the reader. Some attempts at complex cohesive devises, albeit not always successful. |
|  | 2 | Unsatisfactory use of cohesive devices resulting in occasional strain on reader. |
|  | 1 | Cohesion is almost absent causing a frequent strain on reader. Or not enough to evaluate. |
| Vocabulary | 4 | Sophisticated range with effective word/idiom choice and usage. Consistently appropriate register. |
|  | 3 | Adequate range with occasional errors of word/idiom choice, but meaning is not obscured. Mostly appropriate register. |
|  | 2 | Limited range with frequent errors of word/idiom choice. Meaning is confused or obscured. Frequently inappropriate register. |
|  | 1 | Very limited range of vocabulary and idioms. Consistently inappropriate register. Or not enough to evaluate. |
| Language use | 4 | Sentence construction is varied and elaborate. No errors of agreement, tense, number, word order, articles, pronouns or prepositions. |
|  | 3 | Simple sentence structure, some attempts at complex constructions albeit not always successful. Occasional errors but meaning is not obscured. |
|  | 2 | Major problems also in simple sentence constructions with little attempt at complex constructions and frequent errors. Meaning is confused or obscured. |
|  | 1 | Virtually no mastery of sentence construction rules and a text dominated by errors. Or not enough to evaluate. |
| Spelling | 4 | Mastery of conventions, no errors. |
|  | 3 | Occasional errors, but meaning is not obscured and no strain on reader. |
|  | 2 | Frequent errors of spelling. Meaning is confused or obscured and strain on reader. |
|  | 1 | No mastery of conventions. Text is dominated by errors. Or not enough to evaluate. |
| Punctuation | 4 | Mastery of conventions with no errors in punctuation and capitalisation. Occasional errors in the use of commas are allowed. |
|  | 3 | Occasional errors of punctuation and capitalisation, but meaning is not obscured. No strain on reader |
|  | 2 | Frequent errors of punctuation and capitalisation, meaning is confused or obscured. Strain on reader |
|  | 1 | No mastery of conventions. Text dominated by errors of punctuation and capitalisation. Or not enough to evaluate |
