# Supplementary material for: Self-efficacy for writing and written text quality of upper secondary students with and without reading difficulties
Source: Front Psychol. 2023 Sep 22;14:1231817. doi: 10.3389/fpsyg.2023.1231817 (PMC10557487; doi:10.3389/fpsyg.2023.1231817)
Supplement: Supplementary file 2 [file Table_2.docx]

# Supplementary Table 2

*Prompts and Accompanying Self-Efficacy for Writing Statements Used in the Web Survey*

*Rate how sure you are that you can do the things described below when you*

*write in Swedish.*

*Give your answers on a scale of 1-100 where*

*0 = cannot do it at all*

*100 = completely sure/confident that I can do it*

*Write your responses in the boxes to the right. All boxes must be filled in with a*

*number from 0 to 100.*

| Self-efficacy for writing statements |
| --- |
| 1. I can think of many ideas for my writing 2. I can transform my ideas into written text 3. I can think of many words to describe my ideas 4. I can come up with many new ideas 5. I know exactly how to organise my ideas into my writing 6. I can spell my words correctly 7. I can write complete sentences 8. I can punctuate correctly, i.e., put punctuation marks such as full stop and comma, etc., in my sentences 9. I can write grammatically correct sentences 10. I can begin my paragraphs in the right spots 11. I can focus on my writing for at least one hour 12. I can ignore distractions while I’m writing 13. I can start writing assignments quickly 14. I can control my frustration while I’m writing 15. I can think of my writing goals before I write 16. I can keep writing even when it’s difficult |
